# The utilization of seawater for the hydrolysis of macroalgae and subsequent bioethanol fermentation

**DOI:** 10.1038/s41598-020-66610-9

**Published:** 2020-06-16

**Authors:** Darren Greetham, Jessica M. Adams, Chenyu Du

**Affiliations:** 1 School of Applied Sciences, University of Huddersfield, Queensgate, Huddersfield HD1 3DH United Kingdom; 2 IBERS, Aberystwyth University, Gogerddan, Aberystwyth, Ceredigion SY23 3EE United Kingdom

**Keywords:** Biofuels, Chemical modification

## Abstract

A novel seawater-based pretreatment process was developed to improve the hydrolysis yield of brown (*Laminaria digitata)*, green (*Ulva linza)* and red (*Porphyra umbilicalis*) macroalgae. Pre-treated with 5% sulphuric acid at 121 °C, 15 minutes, *L. digitata, U. linza* and *P. umbilicalis* liberated 64.63 ± 0.30%, 69.19 ± 0.11% and 63.03 ± 0.04% sugar in seawater compared with 52.82 ± 0.16%, 45.93 ± 0.37% and 48.60 ± 0.07% in reverse-osmosis water, respectively. Low hydrolysis yields (2.6–11.7%) were observed in alkali and hydrothermal pretreatment of macroalgae, although seawater led to relatively higher yields. SEM images of hydrolyzed macroalgae showed that reverse-osmosis water caused contortions in the remaining cell walls following acid and hydrothermal pre-treatments in the *L. digitata* and *U. linza* samples. Fed-batch fermentations using concentrated green seaweed hydrolysates and seawater with marine yeast *Wickerhamomyces anomalus* M15 produced 48.24 ± 0.01 g/L ethanol with an overall yield of 0.329 g/g available sugars. Overall, using seawater in hydrolysis of seaweed increased sugar hydrolysis yield and subsequent bioethanol production.

## Introduction

The worldwide demand of renewable energy directive has led to a focus on the production of sustainable biofuels. Use of arable land for biofuel has become associated with adverse environmental impacts^1^, resulting in rising food prices and has limited the expansion of the bioethanol industry^2^. Besides the issues associated with land usage, significant quantities of fresh water are used during bioethanol production. It has been estimated that the average global water footprint for bioethanol production is around 2855 L H_2_O per L EtOH^3^. With increasing concerns about water shortage, the availability of fresh water has become a further barrier limiting bioethanol production. As a consequence, biofuel production using marine biomass, such as seaweed is becoming increasingly attractive.

Macroalgae are a promising feedstock for the production of bioethanol, since they do not require fresh water to grow. In addition, they are available in abundance, are not considered a major food source in Europe, do not occupy arable land or require fertilisers to grow^4^. The UK is a recognised centre for macroalgae biodiversity with around 644 different species inhabiting its coastal waters^5^. Despite this abundance and diversity, harvested quantities are currently low (~3000 tonnes dry weight annually)^6^. This is largely as a result of distribution and accessibility concerns and limitations on harvesting and licensing. There is a paucity of information on the economics of macroalgae production and the information tends to cover various species, methods and environmental conditions. There have been several studies on macroalgae farming in tropical conditions^7^, however, these reports are not comparable with production in the North Sea. Feasibility studies have revealed that macroalgae production from the North Sea would be between $155 and $16, 630 per ton dry material depending on the technology used^8^.

Similarly, to lignocellulosic material, macroalgae can be pre-treated and saccharified into fermentable sugars. Various pre-treatment methods have been investigated, such as dilute acid^9^, alkali^10^, hydrothermal^11^ and microwave^12^ pre-treatment. However, the sugar recovery yield from macroalgae was relatively low, ranging from 7.2–61.2%^13,14^. As a result, the sugar concentration in the hydrolysate was typically less than 60 g/L. This significantly limits the potential for bioethanol production, leading to a typical bioethanol concentration of less than 30 g/L^15^. In order to fully utilize the sugars in the macroalgae, enzymatic treatment using cellulase, β-glucosidase^16^ and/or alginate lyase^17^ were explored following physiochemical hydrolysis. Improved sugar recovery yields of 63.1% in red seaweed *G. verrucosa*;^18^ 81.5% in red seaweed *E. cottonii*;^19^ and 84.1% in brown seaweed *L. digitata*^20^ were obtained. In comparison with physicochemical hydrolysis, enzymatic hydrolysis requires significantly longer treatment time (e.g. 48 hours) and the usage of expensive enzymes. Therefore, there are additional costs which would have to be spent on the process. Compared with the 1^st^ and 2^nd^ generation bioethanol production processes, seaweed hydrolysis yield is much lower than that achieved in the hydrolysis of starch materials and cellulosic biomass^21^, indicating further improvement in seaweed hydrolysis is required.

In this study, a novel seawater based seaweed hydrolysis process was explored. Three algae species, *Laminaria digitata*, an abundant brown seaweed (*Phaeophyta*), *Ulva linza*, a green seaweed (*Chlorophyta*) and *Porphyra umbilicalis* an edible red algae (*Rhodophyta*) were investigated for the hydrolysis and subsequent bioethanol fermentation. The impact of salt (in the seawater) on the changes in algae morphology during the hydrolysis was examined using SEM. Post pre-treatment hydrolysates were fermented using a marine-derived yeast *Wickerhamomyces anomalus* M15 which has previously been shown to be salt and inhibitor tolerant to maximise ethanol production^22^.

## Materials and Methods

### Microorganism

*Wickerhamomyces anomalus* M15 was isolated from the marine environment^22^. This yeast was maintained on YPD (2% glucose, 2% peptone, and 1% yeast extract prepared using reverse osmosis water (RO)) as described in^22^.

### Seawater and macroalgae collections

Seawater used for the fermentations was collected from Skegness, UK in June, 2017 and was taken from approximately five metres from the shore and at a depth of one metre. Seawater was allowed to sediment for approximately 24 hours before being filtered through Whatman glass microfiber filters (pore size, 1.2 µm). After filtration, the seawater was autoclaved at 121 °C for 15 min. Sterilized seawater was then stored at 4 °C till required. Salinity was measured using an Elite CTS Tester (ThermoFisher, UK) with the samples diluted until the reading could be compared against a calibration curve (salt concentrations 0.01–0.1%) and then multiplied by the dilution factor to give the correct reading.

*L. digitata*, *U. linza*, and *P. umbilicalis* were collected at Aberystwyth, Cardigan Bay, Wales, UK, in July 2018, frozen within 2 hours of collection. Material was then placed in a Super Modulyo freeze-drier (Edwards, now Cole-Parmer, Cambridgeshire, UK) for seven days to remove all moisture from the samples before the bags were zip-locked closed with minimal air and sent by courier to the University of Huddersfield. The macroalgae were ground using a rotary blender to approximate particle size of less than 0.11 mm, and dried in an oven at 60 °C until constant weight.

### Total carbohydrate analysis

30 mg dried seaweed was subjected to 1 mL 12 M H_2_SO_4_ and the contents incubated at 37 °C in a water bath for 1 hour. 11 mL of water was added to dilute the acid concentration to 1 M and the contents were further incubated for 2 hours^23^. The concentration of monosaccharides was quantified as described in the HPLC section.

### Pre-treatment processing

Three pre-treatments methods were conducted in this study, on all three macroalgae types, with solutions prepared in both RO water and filtered seawater. All assays were performed in triplicate.

i) The dilute acid pre-treatment: macroalgae powder was suspended in 1–5% sulphuric acid (prepared with either RO or seawater as appropriate) at a solid load ratio of 10% (w/v) and autoclaved at 121 °C for 15 minutes. After autoclaving, the biomass was filtered using muslin cloth and a Buchner funnel, and the hydrolysates stored at 4 °C until further use. The solid fraction was further dried at 30 °C for 3 days until dry enough to be examined by SEM.

ii) The alkaline pre-treatments^24^: macroalgae powder was suspended in 5% NaOH at a solid load ratio of 10% (w/v) and incubated in a 50 °C water bath for 12 hours, at 180 rpm. After pre-treatment, the hydrolysate was filtered as above and stored at 4 °C until further use. The wet residue was dried overnight at 30 °C and examined by SEM.

iii) The hydrothermal pre-treatments: macroalgae powder was suspended in RO water or seawater at a solid load ratio of 10% (w/v) and were autoclaved at 121 °C for 15 minutes. After autoclaving, the biomass was filtered as above and the hydrolysates stored at 4 °C until further analysis. The wet residue was dried overnight at 30 °C and examined by SEM.

### Concentrating hydrolysates derived from green seaweed

Hydrolysates were derived from green seaweed following a 5% sulphuric acid and RO water pre-treatment was concentrated using a rotary evaporator at 60 °C, 60 rpm for 2 hours. Approximately 250 mL of seaweed hydrolysate was loaded each time, and approximately 18 mL concentrated hydrolysate was collected.

### Pre-treatments with synthetic seawater and salt solutions

*U. linza* was pre-treated with 1% sulphuric acid using RO water, seawater and synthetic seawater (SW x1), synthetic seawater with twice the reported salt concentrations (SW x 2) and salt solutions based on their presence in seawater. SW x 1 contained 2.7% NaCl, 0.33% MgSO_4_, 0.25% MgCl_2_, 0.1% CaCl_2_, and 0.07% KCl, and SW x 2 contained 5.4% NaCl, 0.66% MgSO_4_, 0.5% MgCl_2_, 0.2% CaCl_2_, and 0.14% KCl, all made up in RO water. Additionally, trials were performed using 2.7, 3.5 or 6% NaCl, 0.33% MgSO_4_, 0.25% MgCl_2_, 0.1% CaCl_2_, or 0.07% KCl all made in RO water, respectively. To explore the possibility that total salt content was the determining factor in the pre-treatment, assays with 3.5% NaCl, MgSO_4_, MgCl_2_, CaCl_2_, and KCl, respectively were performed.

### Scanning electron microscopy

A small amount of macroalgae was fixed on a specimen stub using double-sided carbon tape. The sample was then coated with gold using a Quorom sc7620 sputter coating machine. The scanning electron micrographs were captured using a JEOL JSM-6060LV scanning electron microscopes with back scatter detector, high vacuum mode, working distance 19 mm, spot size 50.

### Fermentation of macroalgae hydrolysate

Fermentations were carried out using *Wickerhamomyces anomalus* M15 in 100 mL mini-fermentation vessels (MFV) (Wheaton glass bottles, Sigma-Aldrich, US). A single colony from YPD agar plates (48 hours, 30 °C) was inoculated into 5 mL YPD broth, and incubated in a shaking incubator shaker at 30 °C, 200 rpm for 24 hours. The inoculum was transferred into a 500 mL conical flask containing 200 mL YPD medium, then cultivated at 200 rpm, 30 °C, 48 hours. Cells were harvested by centrifuge at 1200 x g 5 mins and washed three times using RO water and then re-suspended in 5 mL RO water. The inoculation ratio was 15 million cells per mL fermentation media. The culture volume was 100 mL in 250 mL shaking flask. The control fermentation medium contained 6% glucose, 2% peptone, 1% yeast extract. The seaweed hydrolysate fermentation medium contained only seaweed hydrolysate without any addition. In all experiments, micro-aerophilic conditions were achieved using a sealed butyl plug and aluminium caps (Fisher Scientific). A hypodermic needle attached with a Bunsen valve was pushed through the rubber septum to enable the release of CO_2_. All experiments were performed in triplicate. Fermentations were conducted at 30 °C unless stated otherwise, at 200 rpm. In the fermentations with different initial pH, the pH was adjusted with 5 M NaOH.

Fermentations using concentrated green seaweed hydrolysate started with approximately 50 g/L total sugars (27.9 glucose, 2.1 galactose, 15.21 xylose, 4.18 arabinose, 0.4 fucose, and 0.1 rhamnose g/L, respectively) with 1% peptone and adjusted to pH 5 using NaOH. *W. anomalus* cells were prepared as described above were inoculated at 1.5 × 10^7^ cells/mL and the fermentations set-up as described previously. An additional 50 g/L total sugars was added after 72 and 144 hours, respectively and samples were collected regularly throughout the experiment. The experiment was carried out in triplicates.

### Sugars and inhibitory compounds analysis using HPLC

Samples were diluted to an expected quantification of 10–50 ppm and analysed using a Dionex ICS3000 HPLC machine using a Dionex CarboPac PA20 3 × 150 mm analytical column. 200 mM and 10 mM sodium hydroxide solutions were used as solvents. Glucose, xylose, fucose, galactose, rhamnose and arabinose were measured and compared to standards (10–100 ppm). Mannitol was determined using a Jasco HPLC system as described in^25^. Briefly, samples were diluted with 5 mM H_2_SO_4_ containing 5 mM crotonic acid as an internal standard. The samples were filtered using a 0.45 μm filter (Millex-HV, Millipore, USA) and then analyzed using a Resex ROA-organic acid H^+^ column at 35 °C using a Refractive Index detector (Jasco). The mobile phase was 5 mM H_2_SO_4_, the flow rate was 0.6 mL/min.

Inhibitor compounds were analysed using a Jasco HPLC system using an Aminex HPX-87H, 300 mm long with 7.8 mm internal diameter column pre-heated to 55 °C. The mobile phase was 5 mM H_2_SO_4_ and was pumped at 0.6 mL/min. Standard dilutions of furfural and hydroxy-methyl furfural (HMF) were run concurrently and were used to generate standard curves of these compounds.

### Ethanol analysis using Gas Chromatography (GC)

The ethanol content in the fermentation samples was analysed using gas chromatography (Bruker CP 3900 Agilent, CA, US) as described by^26^. Briefly, the fermentation samples were centrifuged at 1,200 × g 20 °C for 5 minutes. Then the supernatant was diluted 1:100 in RO water and filtered using a 0.45 µm syringe-filter. Helium was used as a carrier gas at a flow rate of 1.2 mL/min. The temperatures of injector and interface were 250 °C and 280 °C, respectively. The following temperature program was used for the column oven: 70 °C for 2 min, a linear ramp to 250 °C at 10 °C/min, held at 250 °C for 5 min. The electron impact (EI)-ionization was performed at 70 eV. The injection volume was 10 µL.

### Statistics

Microsoft Excel was used for the calculation of means and standard deviations. Multivariate clustering was determined using Minitab 18.1. Student’s *t*-Test was carried out and p-value <0.05 was considered to be significant.

## Results and Discussion

### Total carbohydrate analysis

The total carbohydrate analysis following an acid digestion on the three types of seaweed was determined. As shown in Table 1, *L. digitata* contained 249.4 ± 1.2 mg/g, *U. linza* 292.0 ± 1.4 mg/g and *P. umbilicalis* 291.0 ± 0.9 mg/g, respectively (Table 1). There is considerable variation in carbohydrate content for all seaweed types, brown seaweeds have been reported to contain 21.7–68.1%, green seaweeds 23.8–59%, and red seaweeds 21.8–75.7%, respectively (Table 2). Research has shown considerable seasonal variation in carbohydrate content for the same seaweed in the same location^17,27^. Mannitol made up 12.3% of the dry weight of *L. digitata*, which is within the expected range of mannitol content for *L. digitata* harvested in May-June from the Welsh coast^17^.

**Table 1:** The total carbohydrate content of *L. digitata*, *U. linza*. *P. umbicalis* (mg/g).

	Glucose	Xylose	Arabinose	Galactose	Fucose	Rhamnose	Mannitol	Total Sugar
**Seaweed**								
*L. digitata*	20.3 ± 0.2	12 ± 0.0	3.1 ± 0.2	12.0 ± 0.7	42.0 ± 0.2	37.0 ± 0.0	123 ± 0.6	249.4 ± 1.2
*U. linza*	131 ± 3.0	88 ± 3.0	28 ± 0.1	27.0 ± 0.1	16.0 ± 0.2	1.00 ± 0.0	1.0 ± 0.0	292.0 ± 1.4
*P. umbilicalis*	103 ± 0.2	59 ± 0.2	7.0 ± 0.03	101 ± 0.3	18.0 ± 0.1	2.00 ± 0.0	1.0 ± 0.0	291.0 ± 0.9

**Table 2:** The total carbohydrate content of seaweeds, total sugar concentration in the hydrolysate and sugar recovery yield (%, w/w).

Seaweed	Total Carbohydrate (%)	Hydrolysis method and conditions	Total liberated monosaccharides (g/L)	Recovery Yield (%)	Reference
**Brown seaweeds**					
*Laminara digitata*	24.6	Seawater, 1:10 (SLR), 5% H_2_SO_4_, 121 °C, 15 mins	20.81	64.6	This study
*Laminara digitata*	21.7	1:10 (SLR), 5% H_2_SO_4_, 121 °C, 15 mins	8.48	39.0	^13^
*Laminara digitata*	18.3	Cellulase and alginate lyase, 50 °C, 48 h	14.11	84.1	^20^
*Laminara digitata*	68.1	3:100 (SLR), 5% H_2_SO_4_, 135 °C, 15 mins		11.1	^39^
*Fucus serratus*	26.4	1:10 (SLR), 5% H_2_SO_4_, 121 °C, 15 mins	11.09	42.0	^13^
*Sargassum latifolium*	20.1	1:10 (SLR), 1% H_2_SO_4_, 121 °C, 60 mins	1.45	7.2	^14^
*Undaria pinnatifida*	45.7	1:7.6 (SLR), 0.4% H_2_SO_4_, 121 °C, 60 mins	33.1	50.1	^41^
*Undaria pinnatifida*	19.5	N/A	N/A	N/A	^27^
*Sacc polyschides*	17.6	N/A	N/A	N/A	^27^
*Sargassum muticum*	16.6	N/A	N/A	N/A	^27^
*Saccharina latissima*	22.3	N/A	N/A	N/A	^27^
*Him elongata*	35.5	N/A	N/A	N/A	^27^
**Green seaweeds**					
*Ulva linza*	29.2	1:10 (SLR), 5% H_2_SO_4_, 121 °C, 15 mins	26.2	69.1	This study
*Ulva lactuca*	23.8	1:10 (SLR), 5% H_2_SO_4_, 121 °C, 15 mins	11.91	50.0	^13^
*Ulva lactuca*	19.8	1:10 (SLR), 1% H_2_SO_4_, 121 °C, 60 mins	1.4	7.4	^14^
*Ulva lactuca*	25.8	Enzymatic	10.71	41.5	^43^
*Ulva reticulata*	18.0	N/A	N/A	49.0	^44^
*Ulva prolifera*	58.5	0.2% hydrogen peroxide, 50 °C, pH 4.0, 12 h	5.9	10.0	^45^
*Codium tomentosum*	15.9	N/A	N/A	N/A	^27^
*Ulva lactuca*	31.4	N/A	N/A	N/A	^27^
**Red Seaweeds**					
*Palmaria umbicalis*	29.1	1:10 (SLR), 5% H_2_SO_4_, 121 °C, 15 mins	24.0	63.0	This study
*Chondrus crispus*	21.8	1:10 (SLR), 5% H_2_SO_4_, 121 °C, 15 mins	13.3	61.2	^13^
*Palmaria palmata*	39.4	1:10 (SLR), 5% H_2_SO_4_, 121 °C, 15 mins	16.1	40.9	^13^
*Jania rubens*	11.6	1:10 (SLR), 1% H_2_SO_4_, 121 °C, 60 mins	1.10	9.48	^14^
*Gel sesquipedale*	30.9	Enzymatic	6.68	21.6	^43^
*Gelidium amansii*	62.6	1:8 (SLR), 3.6% H_2_SO_4_, 142.6 °C, 11 mins.	45.1	57.6	^40^
*Gelidium amansii*	62.8	1:8 (SLR), 182 mM, 121 °C, 45 mins.	35.9	45.7	^46^
*Kappaphucus alvarezii*	71.7	1:8 (SLR), 1.8% H_2_SO_4_, 121 °C, 45 mins.	30.6	30.9	^38^
*Eucheuma cottonii*	75.7^a^	16% (w/v) loading ratio, solid acid 120 °C, 1 h, then cellulase and β-glucosidase, 50 °C, 30 h	98.7	81.5	^19^
*Grac verrucosa*	53.5	1:12 (SLR), 2.7% H_2_SO_4_, 121 °C, 60 mins	39.6	74.0	^47^
*Eucheuma spinosum*		1:5 (SLR), 2.8% H_2_SO_4_, 121 °C, 60 mins	44.2	49.6	^42^
*Gelidium amansii*	74.4	1:12 (SLR), 0.9% H_2_SO_4_, 121 °C, 45 mins	25.6	41.2	^48^
*Gracilaria verrucosa*	56.6	1:20 (SLR), 5% NaOH, 90 °C, 2 h, then enzymatic saccharification		63.1	^18^
*Gracilaria verrucosa*	29.1	N/A	N/A	N/A	^27^
*Palmaria palmata*	36.9	N/A	N/A	N/A	^27^
*Asp armata*	16.9	N/A	N/A	N/A	^27^
Seaweed mixture ^b^	40.8	1:12 (SLR), 2.9% H_2_SO_4_, 121°C, 90 mins	9.7	28.5	^49^

^a^carbohydrate content in the macroalgae extract. ^b^Red seaweeds - *Gracilaria verrucosa, Ahnfeltiopsis flabelliformis, Grateloupia elliptica, Lomentaria catenata*, and *Hypnea charoides*, Brown seaweeds - *Hizikia fusiformis, Sargassum fulvellum*, and *Undaria pinnatifida* and Green seaweeds - *Ulva intestinalis and Ulva pertusa*. SLR: solid loading ratio (w/v).

### Effect of pre-treatment and water sources on release of monosaccharides from macroalgae

#### Dilute acid pre-treatment

In dilute acid pre-treatments using sulphuric acid (1–5%), higher concentrations of total sugars were obtained in the hydrolysis using seawater than those using RO water (Table 3). Additionally, hydrolysates produced using seaweed and RO water had a salinity of 0.83 ± 0.08%, and those produced using seaweed and seawater had a salinity of 4.1 ± 0.1%.

**Table 3:** Composition of sugars in macroalgae hydrolysates following pre-treatment. RO = reverse osmosis water, SW = seawater. N = 3, values show ± standard deviation.

	Glucose	Xylose	Arabinose	Galactose	Fucose	Rhamnose	Mannitol	Total sugars	Hydrolysis yield (%)
**Dilute acid pre-treatment**									
1% sulphuric acid									
*L. digitata* (RO)	1.32 ± 0.07	0.62 ± 0.04	0.16 ± 0.06	0.55 ± 0.06	2.39 ± 0.11	0.27 ± 0.07	9.62 ± 0.11	14.93 ± 0.55	36.59 ± 0.22
*L. digitata* (SW)	1.98 ± 0.09	0.67 ± 0.08	0.27 ± 0.05	0.70 ± 0.03	2.48 ± 0.02	0.31 ± 0.01	11.4 ± 0.54	17.77 ± 0.64	47.02 ± 0.34
*U. linza sp* (RO)	8.16 ± 0.09	5.51 ± 0.08	2.27 ± 0.05	0.67 ± 0.03	0.90 ± 0.02	0.02 ± 0.01	0	16.74 ± 0.09	36.20 ± 0.10
*U. linza sp* (SW)	8.81 ± 0.13	6.01 ± 0.07	2.54 ± 0.08	0.71 ± 0.03	0.80 ± 0.02	0.02 ± 0.01	0	18.20 ± 0.15	41.80 ± 0.13
*P. umbilicalis* (RO)	3.52 ± 0.16	2.95 ± 0.24	0.32 ± 0.08	6.29 ± 0.24	1.25 ± 0.09	0.02 ± 0.02	0	14.35 ± 0.38	29.73 ± 0.17
*P. umbilicalis* (SW)	5.01 ± 0.24	3.53 ± 0.31	0.52 ± 0.04	6.92 ± 0.31	1.33 ± 0.12	0.04 ± 0.04	0	17.35 ± 0.55	38.53 ± 0.12
2% sulphuric acid									
*L. digitata* (RO)	1.78 ± 0.08	0.65 ± 0.03	0.18 ± 0.02	0.59 ± 0.09	2.58 ± 0.07	0.27 ± 0.02	10.43 ± 0.64	16.48 ± 0.58	39.29 ± 0.50
*L. digitata* (SW)	1.99 ± 0.01	0.89 ± 0.04	0.28 ± 0.12	0.91 ± 0.04	2.99 ± 0.12	0.32 ± 0.03	12.24 ± 0.70	19.64 ± 0.67	61.52 ± 0.08
*U. linza* (RO)	8.59 ± 0.12	5.78 ± 0.09	2.41 ± 0.08	0.68 ± 0.03	0.91 ± 0.01	0.02 ± 0.00	0	18.39 ± 0.09	36.94 ± 0.11
*U. linza* (SW)	10.58 ± 0.2	7.89 ± 0.17	2.68 ± 0.11	0.72 ± 0.09	0.95 ± 0.01	0.02 ± 0.00	0	22.85 ± 0.31	55.05 ± 0.33
*P. umbricalis* (RO)	3.91 ± 0.21	3.01 ± 0.23	0.35 ± 0.02	6.58 ± 0.08	1.33 ± 0.18	0.02 ± 0.00	0	15.20 ± 0.21	30.63 ± 0.26
*P. umbricalis* (SW)	5.81 ± 0.11	4.18 ± 0.31	0.56 ± 0.03	7.49 ± 0.08	1.55 ± 0.23	0.03 ± 0.00	0	19.62 ± 0.74	52.03 ± 0.07
**3% sulphuric acid**									
*L. digitata* (RO)	2.01 ± 0.01	0.81 ± 0.02	0.20 ± 0.00	0.65 ± 0.03	2.78 ± 0.34	0.28 ± 0.04	11.04 ± 0.74	17.77 ± 0.79	44.57 ± 0.12
*L. digitata* (SW)	1.98 ± 0.03	1.13 ± 0.05	0.22 ± 0.03	1.01 ± 0.13	3.49 ± 0.25	0.32 ± 0.03	12.26 ± 0.28	20.41 ± 0.34	67.69 ± 0.13
*U. linza* (RO)	9.99 ± 0.25	6.88 ± 0.19	2.58 ± 0.28	0.73 ± 0.06	0.93 ± 0.01	0.02 ± 0.00	0	21.12 ± 0.74	40.57 ± 0.30
*U. linza* (SW)	12.2 ± 0.37	8.29 ± 0.56	3.01 ± 0.17	0.76 ± 0.10	1.08 ± 0.00	0.02 ± 0.00	0	25.36 ± 0.67	61.94 ± 0.25
*P. umbricalis* (RO)	4.86 ± 0.20	4.89 ± 0.24	0.37 ± 0.01	7.69 ± 0.49	1.33 ± 0.21	0.02 ± 0.00	0	19.14 ± 0.51	35.30 ± 0.17
*P. umbricalis* (SW)	6.12 ± 0.26	5.28 ± 0.55	0.58 ± 0.09	7.59 ± 0.23	1.59 ± 0.18	0.02 ± 0.00	0	21.18 ± 0.80	59.49 ± 0.27
**5% sulphuric acid**									
*L. digitata* (RO)	2.02 ± 0.01	0.83 ± 0.02	0.19 ± 0.00	0.67 ± 0.03	3.80 ± 0.23	0.29 ± 0.01	12.22 ± 0.01	20.00 ± 0.25	52.82 ± 0.16
*L. digitata* (SW)	2.00 ± 0.01	1.19 ± 0.01	0.30 ± 0.00	1.10 ± 0.04	3.67 ± 0.30	0.33 ± 0.00	12.24 ± 0.00	20.81 ± 0.36	64.63 ± 0.30
*U. linza* (RO)	11.01 ± 0.3	6.98 ± 0.21	2.58 ± 0.19	0.72 ± 0.03	1.19 ± 0.00	0.02 ± 0.00	0	22.49 ± 0.73	45.93 ± 0.04
*U. linza* (SW)	12.9 ± 0.11	8.41 ± 0.24	2.76 ± 0.03	0.76 ± 0.03	1.38 ± 0.00	0.02 ± 0.00	0	26.23 ± 0.23	69.19 ± 0.11
*P. umbricalis* (RO)	5.38 ± 0.3	4.91 ± 0.16	0.38 ± 0.02	7.69 ± 0.14	1.34 ± 0.17	0.02 ± 0.00	0	19.72 ± 0.45	48.60 ± 0.07
*P. umbricalis* (SW)	8.12 ± 0.35	5.32 ± 0.09	0.61 ± 0.05	8.33 ± 0.12	1.62 ± 0.07	0.02 ± 0.00	0	24.02 ± 0.27	63.03 ± 0.04
**Hydrothermal**									
*L. digitata* (RO)	0.27 ± 0.12	0.15 ± 0.02	0.01 ± 0.001	0.07 ± 0.01	0.35 ± 0.02	0	2.26 ± 0.59	3.11 ± 0.59	0.53 ± 0.04
*L. digitata* (SW)	0.33 ± 0.09	0.18 ± 0.07	0.01 ± 0.005	0.08 ± 0.02	0.38 ± 0.04	0	2.67 ± 0.62	3.65 ± 0.21	1.96 ± 0.16
*U. linza* (RO)	2.15 ± 0.18	1.84 ± 0.23	0.59 ± 0.02	0.02 ± 0.01	0	0	0	4.60 ± 0.15	0.66 ± 0.04
*U. linza* (SW)	2.34 ± 0.23	1.96 ± 0.29	0.55 ± 0.18	0.02 ± 0.01	0	0	0	4.87 ± 0.21	2.19 ± 0.07
*P. umbilicalis* (RO)	1.29 ± 0.21	0.69 ± 0.04	0.02 ± 0.01	1.09 ± 0.02	0.59 ± 0.01	0	0	3.68 ± 0.28	0.53 ± 0.04
*P. umbilicalis* (SW)	1.89 ± 0.34	0.84 ± 0.02	0.02 ± 0.01	1.29 ± 0.07	0.64 ± 0.01	0	0	4.69 ± 0.59	2.12 ± 0.07
**Alkaline**									
*L. digitata* (RO)	0.25 ± 0.09	0.11 ± 0.02	0.01 ± 0.01	0.06 ± 0.01	0.39 ± 0.08	0.02 ± 0.01	3.09 ± 0.15	3.93 ± 0.52	10.22 ± 0.09
*L. digitata* (SW)	0.26 ± 0.05	0.16 ± 0.01	0.01 ± 0.02	0.07 ± 0.02	0.35 ± 0.02	0.04 ± 0.01	3.37 ± 0.24	4.26 ± 0.36	11.79 ± 0.10
*U. linza*(RO)	1.98 ± 0.02	1.94 ± 0.06	0.65 ± 0.06	0.01 ± 0.01	0.02 ± 0.01	0.01 ± 0.00	0.02 ± 0.00	4.63 ± 0.64	10.26 ± 0.14
*U. linza* (SW)	2.09 ± 0.01	2.05 ± 0.02	0.68 ± 0.01	0.01 ± 0.01	0.05 ± 0.01	0.01 ± 0.00	0.03 ± 0.01	4.92 ± 0.23	11.61 ± 0.21
*P. umbilicalis* (RO)	1.19 ± 0.01	0.59 ± 0.04	0.03 ± 0.04	0.98 ± 0.09	0.46 ± 0.08	0.02 ± 0.00	0.04 ± 0.00	3.31 ± 0.34	7.36 ± 0.09
*P. umbilicalis* (SW)	1.29 ± 0.02	0.61 ± 0.05	0.01 ± 0.01	1.02 ± 0.08	0.59 ± 0.08	0.02 ± 0.00	0.05 ± 0.01	3.59 ± 0.21	8.50 ± 0.01

For *L. digitata*, a pre-treatment with 1% sulphuric acid and seawater led to the hydrolysate containing significantly higher concentration of total sugar (17.77 ± 0.64 g/L) than that using RO water (14.93 ± 0.55 g/L, *p* = 0.001). The highest total sugar concentration obtained was 20.81 ± 0.36 g/L when 5% sulphuric acid was used, corresponding to a sugar recovery yield of 64.63 ± 0.30%. Furthermore, presence of acid in the pre-treatment also increased recovery of the liquid fraction after pre-treatment (Fig. S1). For instance, in assays using RO water and no acid, only 20.6 ± 1.1% of the liquid fraction was recovered post hydrolysis, while pre-treatment using seawater resulted in 34.7% ± 2.6% liquid recovered (Fig. S1). Use of seawater and 3% sulphuric acid improved the recovery of the liquid fraction to 81.6 ± 2.0% (Fig. S1).

When determining release of individual substrates after a 1% sulphuric acid pre-treatment, revealed that 62.0 ± 0.4% of glucose, and 60.3 ± 0.4% of mannitol, respectively, had been released (Table 4). This compared with 39.2 ± 0.3% and 47.1 ± 0.0 when using RO water (Table 4). As sulphuric acid concentration increased so did total sugar recovered. Multivariate clustering revealed that the sugar composition of a hydrolysate using 1% sulphuric acid with seawater was similar to the sugar composition of a hydrolysate using 3% sulphuric acid with RO water (data not shown). This suggested that using seawater could reduce the usage of sulphuric acid to achieve a similar hydrolysis yield. When 5% sulphuric acid was used in the hydrolysis of *L. digitata*, 64.63 ± 0.30% total sugar was hydrolysed using seawater while 52.82 ± 0.16% total sugar was hydrolysed using RO water (Table 3).

**Table 4:** Presence of individual sugars in hydrolysates following a pre-treatment as a comparison with theoretical maxima (% of theoretical maxima) for *L. digitata*, *U. linza*, and *P. umbicalis*. N = 3, values show ± standard deviation.

% release compared with theoretical maxima							
	Glucose	Xylose	Arabinose	Galactose	Fucose	Rhamnose	Mannitol
**Dilute acid pre-treatment**							
**1% sulphuric acid**							
*L. digitata* (RO)	39.21 ± 0.3	31.15 ± 0.3	31.12 ± 1.9	27.63 ± 0.5	34.31 ± 0.2	4.41 ± 0.1	47.16 ± 0.0
*L. digitata* (SW)	62.03 ± 0.4	36.08 ± 0.6	56.28 ± 1.6	37.69 ± 0.2	38.16 ± 0.1	5.41 ± 0.0	60.37 ± 0.4
*U. linza sp* (RO)	37.56 ± 0.0	37.75 ± 0.0	48.88 ± 0.1	14.96 ± 0.1	33.91 ± 0.1	12.0 ± 1.0	0
*U. linza sp* (SW)	43.46 ± 0.0	44.13 ± 0.7	58.62 ± 0.2	16.99 ± 0.2	32.31 ± 0.1	12.92 ± 1.0	0
*P. umbilicalis* (RO)	20.60 ± 0.1	30.15 ± 0.4	27.56 ± 0.4	37.55 ± 0.2	41.87 ± 0.5	6.03 ± 1.0	0
*P. umbilicalis* (SW)	31.43 ± 0.2	38.66 ± 0.5	48.00 ± 0.5	44.27 ± 0.3	47.75 ± 0.6	12.92 ± 2.0	0
**2% sulphuric acid**							
*L. digitata* (RO)	51.43 ± 0.3	31.77 ± 0.2	34.05 ± 0.6	28.83 ± 0.7	36.03 ± 0.1	4.28 ± 0.0	49.73 ± 0.6
*L. digitata* (SW)	75.61 ± 0.6	57.20 ± 0.3	69.67 ± 0.3	58.49 ± 0.3	54.91 ± 0.2	6.67 ± 0.0	76.75 ± 0.6
*U. linza* (RO)	38.46 ± 0.0	38.52 ± 0.1	50.48 ± 0.2	14.77 ± 0.1	33.36 ± 0.0	11.73 ± 0.0	0
*U. linza* (SW)	56.40 ± 0.1	60.39 ± 0.2	73.82 ± 0.2	20.56 ± 0.3	45.79 ± 0.0	15.42 ± 0.0	0
*P. umbricalis* (RO)	22.26 ± 0.2	29.92 ± 0.3	29.32 ± 0.2	38.21 ± 0.7	43.34 ± 0.1	5.86 ± 0.0	0
*P. umbricalis* (SW)	43.51 ± 0.2	54.64 ± 0.5	61.70 ± 0.4	57.20 ± 0.8	66.42 ± 0.1	15.42 ± 0.0	0
**3% sulphuric acid**							
*L. digitata* (RO)	61.09 ± 0.5	41.64 ± 0.1	39.80 ± 0.0	33.42 ± 2.0	40.84 ± 0.8	4.66 ± 0.2	55.38 ± 0.6
*L. digitata* (SW)	79.65 ± 1.0	76.90 ± 0.6	57.95 ± 0.9	68.73 ± 1.0	67.86 ± 0.3	7.06 ± 0.3	81.26 ± 0.3
*U. linza* (RO)	42.34 ± 0.2	41.22 ± 0.2	58.61 ± 1.2	16.45 ± 0.0	35.86 ± 0.0	12.34 ± 0.0	0
*U. linza* (SW)	63.77 ± 0.4	67.65 ± 0.7	81.08 ± 1.4	22.68 ± 0.0	55.12 ± 0.0	16.33 ± 0.0	0
*P. umbricalis* (RO)	26.11 ± 0.3	40.68 ± 0.6	32.61 ± 0.3	40.80 ± 0.2	45.59 ± 1.1	6.17 ± 0.0	0
*P. umbricalis* (SW)	48.52 ± 0.6	73.08 ± 0.9	67.66 ± 0.4	61.37 ± 0.2	72.13 ± 1.0	16.33 ± 0.0	0
**5% sulphuric acid**							
*L. digitata* (RO)	67.92 ± 0.5	47.21 ± 0.1	41.83 ± 0.0	38.11 ± 0.2	45.50 ± 0.2	5.34 ± 0.0	52.82 ± 0.0
*L. digitata* (SW)	75.20 ± 0.6	75.69 ± 2.5	73.87 ± 0.3	69.97 ± 0.3	66.70 ± 0.5	6.80 ± 0.0	75.96 ± 0.0
*U. linza* (RO)	47.15 ± 0.4	46.38 ± 0.8	65.33 ± 1.2	18.45 ± 0.1	50.76 ± 0.0	13.65 ± 0.0	0
*U. linza* (SW)	76.33 ± 0.0	72.95 ± 0.9	76.33 ± 0.0	12.48 ± 0.2	65.83 ± 0.0	15.26 ± 0.0	0
*P. umbricalis* (RO)	42.27 ± 0.2	56.80 ± 0.4	37.05 ± 0.2	51.96 ± 0.1	50.81 ± 0.0	6.82 ± 0.0	0
*P. umbricalis* (SW)	60.91 ± 0.7	68.83 ± 0.3	66.52 ± 1.2	62.12 ± 0.4	68.70 ± 0.0	15.26 ± 0.0	0
**Hydrothermal**							
*L. digitata* (RO)	2.74 ± 0.0	2.57 ± 0.1	0.66 ± 0.3	1.20 ± 0.0	1.71 ± 0.0	0	3.78 ± 0.1
*L. digitata* (SW)	6.15 ± 0.3	5.68 ± 0.1	1.21 ± 0.3	2.52 ± 0.1	3.42 ± 0.0	0	8.22 ± 0.1
*U. linza* (RO)	3.38 ± 0.1	4.30 ± 0.2	4.34 ± 0.0	0.15 ± 0.0	0	0	0
*U. linza* (SW)	6.71 ± 0.1	8.44 ± 0.2	7.44 ± 0.6	0.28 ± 0.0	0	0	0
*P. umbilicalis* (RO)	2.57 ± 0.2	2.4 ± 0.0	0.58 ± 0.1	2.22 ± 0.0	6.75 ± 0.0	0	0
*P. umbilicalis* (SW)	6.89 ± 0.3	5.39 ± 0.0	1.08 ± 0.1	4.8 ± 0.0	13.47 ± 0.0	0	0
**Alkaline**							
*L. digitata* (RO)	7.88 ± 0.4	5.86 ± 0.16	2.06 ± 0.32	3.20 ± 0.0	5.94 ± 0.00	0.34 ± 0.7	15.65 ± 0.12
*L. digitata* (SW)	8.82 ± 0.2	9.18 ± 0.0	2.22 ± 0.64	4.01 ± 0.0	5.74 ± 0.01	0.74 ± 0.8	18.87 ± 0.19
*U. linza*(RO)	9.67 ± 0.0	14.10 ± 0.0	14.85 ± 0.2	0.23 ± 0.0	0.08 ± 0.00	6.40 ± 0.0	12.80 ± 0.00
*U. linza* (SW)	10.99 ± 0.0	16.05 ± 0.0	16.73 ± 0.0	0.25 ± 0.0	2.15 ± 0.02	6.89 ± 0.0	20.67 ± 0.00
*P. umbilicalis* (RO)	7.39 ± 0.0	6.40 ± 0.0	2.74 ± 0.51	6.21 ± 0.0	16.35 ± 0.0	6.40 ± 0.0	25.60 ± 0.00
*P. umbilicalis* (SW)	8.62 ± 0.0	7.12 ± 0.0	0.09 ± 0.14	6.95 ± 0.0	22.58 ± 0.0	6.89 ± 0.0	34.45 ± 0.00

The hydrolysis yield obtained in this study for *L. digitata* (64.63 ± 0.3%) was the highest hydrolysis yield reported for brown algae using a thermochemical method. Although this yield was lower than that obtained using enzymatic hydrolysis method (84.1%)^20^, the reaction time for diluted acid method (15 minutes) was significant lower than enzymatic hydrolysis (48 hours) and no expensive enzymes were required.

The overall presence of glucose, arabinose, galactose, and fucose in *L, digitata* samples derived from a 1% sulphuric acid pre-treatment correlated with existing published data^13^. However, there was significantly higher concentrations of mannitol present in hydrolysates derived from *L. digitata*, and the higher concentrations of mannitol where analogous to those observed at peak annual mannitol concentrations in *L. digitata*^17^. The inhibitory compounds such as acetic acid, furfural or phenolic compounds were not detected in the hydrolysates (data not shown).

For *U. linza*, higher concentration of sugars in the hydrolysate was obtained using seawater than those which used RO water (Table 3). A pre-treatment with 1% sulphuric acid in seawater using *U. linza* liberated 41.80 ± 0.13% of available sugars, which compared with 36.20 ± 0.10% when using RO water (*p* = 0.04). The highest sugar content in the hydrolysate was 26.26 ± 0.23 g/L, when 5% sulphuric acid was used in seawater, corresponding to 69.19 ± 0.11% sugar recovery yield. When RO water was used, the sugar content and sugar recovery yield dropped to 22.49 ± 0.73 g/L and 45.93 ± 0.04%, respectively.

For individual sugars, using 1% sulphuric acid in seawater liberated 43.4 ± 0.0% glucose and 44.1 ± 0.7% for xylose, respectively (Table 4). This compared with 37.5 ± 0.3% and 37.7 ± 0.0% when using 1% sulphuric acid with RO water (Table 4). Multivariate clustering revealed that sugar composition of a hydrolysate using 1% sulphuric acid and seawater was similar to the sugar composition of a hydrolysate using 2% sulphuric acid and RO water; and assays using 2% sulphuric acid and seawater clustered together with those using 5% sulphuric acid and RO water (data not shown).

For *P. umbilicalis*, once again there was a higher concentration of sugars in the hydrolysate using seawater when compared with the hydrolysate produced using RO water (Table 3). A pre-treatment with 1% sulphuric acid in seawater on *P. umbilicalis* liberated 38.5 ± 0.1% of available sugars, which compared with 29.7 ± 0.2% when using RO water (*p* = 0.039). Use of 5% sulphuric acid and seawater liberated 24.02 ± 0.27 g/L or 63.0 ± 0.1% available sugars, which compared with 19.68 ± 0.45 g/L or 48.6 ± 0.1% when using RO water (*p* = 0.001). The highest sugar content in the hydrolysate was 24.02 ± 0.27 g//L, when 5% sulphuric acid and seawater was used.

For individual sugars, 1% sulphuric acid in seawater liberated 31.4 ± 0.2% of glucose and 44.2 ± 0.3% of galactose, respectively (Table 4). This compared with 20.6 ± 0.1% and 37.5 ± 0.2% when using 1% sulphuric acid with RO water (Table 4). An additional observation was that after pre-treatment using sulphuric acid in seawater, the *P. umbilicalis* biomass was characterised as a very small particle size when compared with biomass particle size using sulphuric acid in RO water, which may contribute to this increased yield.

#### Hydrothermal pre-treatment

The effect of a hydrothermal pre-treatment of the three macroalgae using either RO or seawater was examined. The sugar concentrations in the hydrolysates were lower than that observed in the dilute acid pre-treatment (Table 3). Approximately 3–4 g/L total sugars were present in the hydrolysate derived from *L. digitata* which compared with 15–21 g/L following a dilute acid pre-treatment and similar was observed for *U. linza* (~4.6–4.87 g/L compared with 18–26 g/L) and *P. umbricalis* (3.6–4.6 g/L compared with 14–24 g/L) (Table 3). Examining for the effect of the presence of seawater, higher sugar contents were obtained in the hydrolysates using seawater when compared with those hydrolysates generated using RO water (Table 3). The low sugar recovery yield (<6%) was mainly due to the significant lower amount of hydrolysate could be collected after the pre-treatment.

#### Alkaline pre-treatment

The effect of an alkaline pre-treatment (5 M NaOH) was studied on the selected macroalgae using either RO or seawater was examined. The sugar contents of hydrolysates were similar to those observed following a hydrothermal pre-treatment (3.3–4.9 g/L), respectively. The hydrolysis yields were significant lower than those following a dilute acid pre-treatment (Table 3).

### Effect of pre-treatment on macroalgae structural morphology

**Figure 1:**
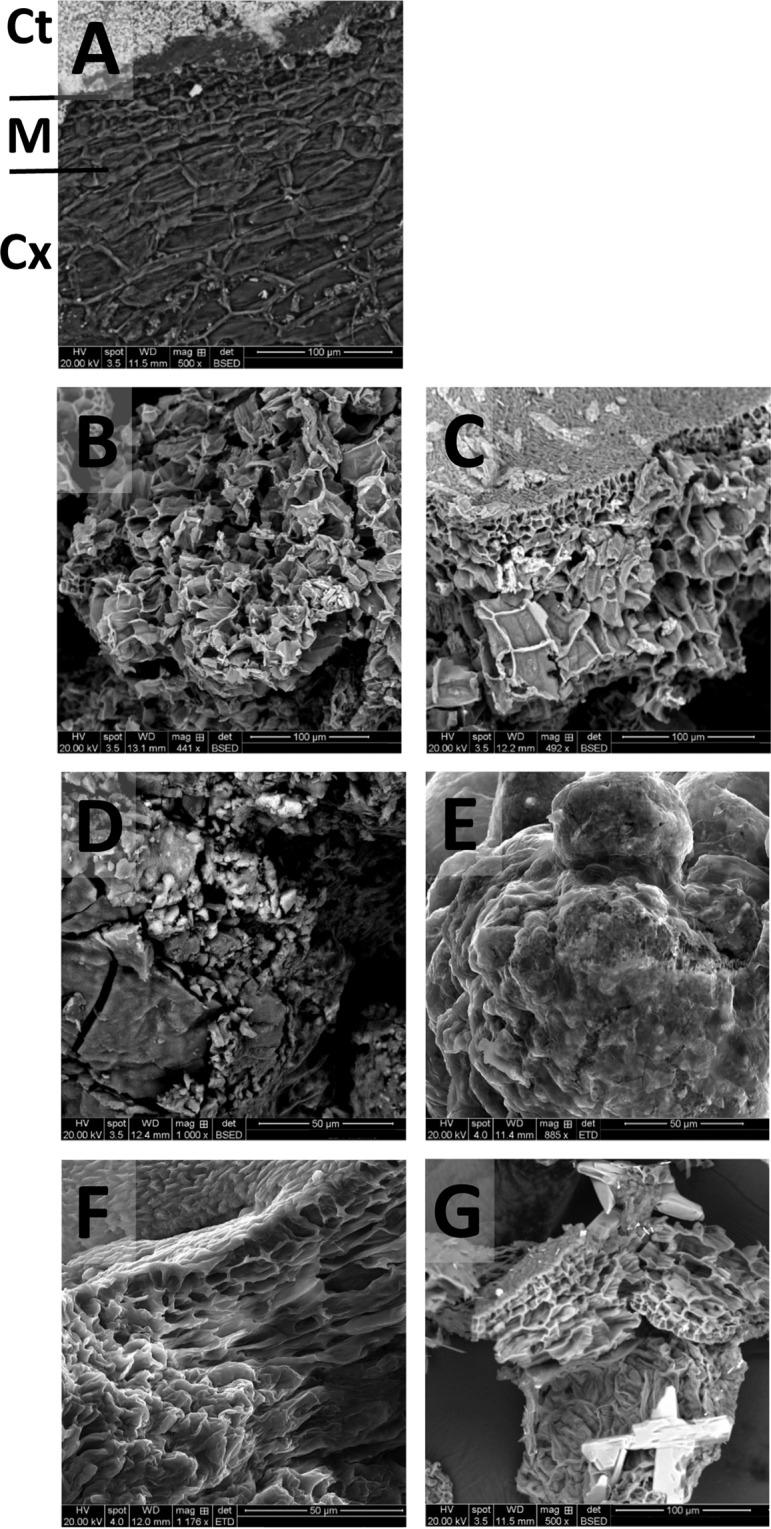
SEM images of *L. digitata* following different thermochemical pre-treatments. (**A**) Freeze-dried sample; (**B**) dilute acid pre-treatment in RO water; (**C**) dilute acid pre-treatment in seawater; (**D**) alkaline pre-treatment in RO water; (**E**) alkaline pre-treatment in seawater; (**F**) hydrothermal pre-treatment in RO water; and (**G**) hydrothermal pre-treatment in seawater.

Scanning Electron Microscopy (SEM) was used in order to investigate the effect of pre-treatment on the macroalgae morphology. Doing this provided an alternative view, giving a better understanding, of the effect of using seawater rather than deionised water on the macroalgal tissues. The dilute acid pre-treatment removed the external cell wall from the *L. digitata* and the *U. linza* samples, as did the hydrothermal pre-treatment for *L. digitata*. Figure 1A shows a cross-section of the freeze-dried *L. digitata* from the external cuticula, (Ct) through the meristoderm (M) and down to the cortex (Cx), where the cells are larger and wider than those closer to the surface. Figure 1B,C show similar tissue structure following a dilute acid pre-treatment, though the external cell walls have been lost. Remaining cell walls in 1B, prepared with RO water, appear more contorted and fluted than those in 1 C, which was prepared with seawater and maintained the original cellular structure within its remaining cell walls. Figure 1D,E show *L. digitata* tissue following alkali pre-treatments, with 1D prepared in RO water and 1E in seawater. Both tissues are morphologically different to the freeze-dried material (1 A), becoming mainly flat (1D) with three-dimensional stacks across it (1E). Figure 1F,G show tissue following hydrothermal pre-treatment, with the overall cellular structure maintained as with the dilute acid pre-treatment, though as before the external cell walls have been lost. Here, the osmotic impact of RO water (1 F) has again caused torsion on the remaining cell walls and visibly altered their shape, compared to those produced with seawater (1 G). Figure 1G also contains a salt crystal within the lower section of the image. Due to the loss of cellular integrity, the remaining cell walls were more susceptible to stress such as osmotic pressure, which is likely to have caused the contortions in the remaining cell walls processed with acid in RO water. The seawater, being isotonic to the cells, did not alter them in this fashion. This is seen in many of the seawater images, which will have occurred during SEM preparation.

**Figure 2:**
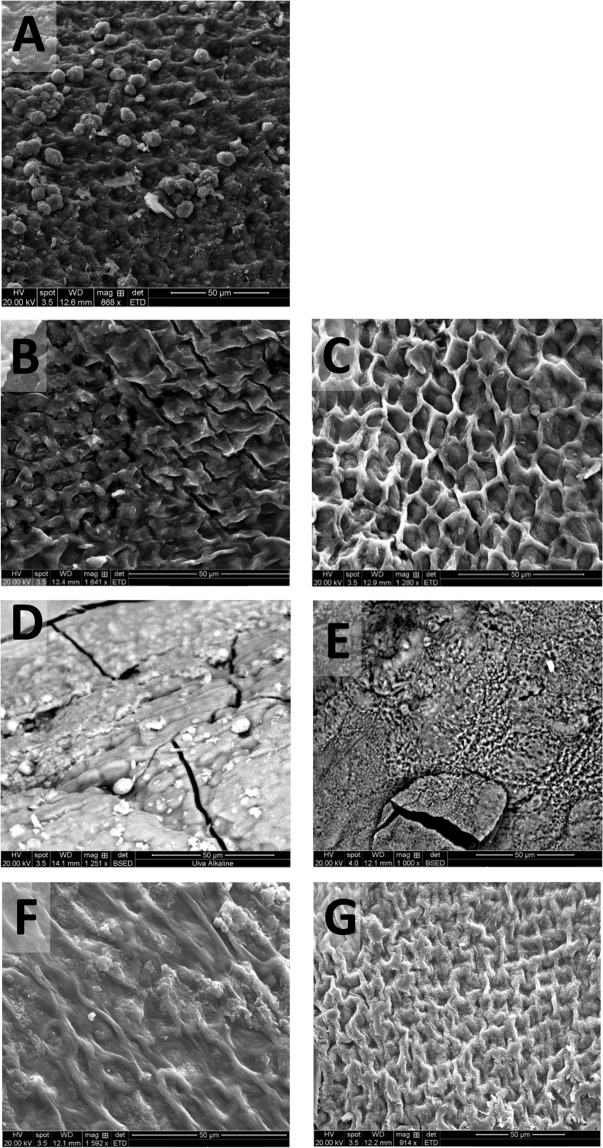
SEM images of *U. linza* following different thermochemical pre-treatments. (**A**) Freeze-dried sample; (**B**) dilute acid pre-treatment in RO water; (**C**) dilute acid pre-treatment in seawater; (**D**) alkaline pre-treatment in RO water; (**E**) alkaline pre-treatment in seawater; (**F**) hydrothermal pre-treatment in RO water; and (**G**) hydrothermal pre-treatment in seawater.

SEM images of *U. linza*, are shown in Fig. 2. The freeze-dried sample in 2 A shows indentations of the outer cell walls to the cells within and some artefacts fixed to the external layer possibly of fine sand grains. Because *U. linza* is two cells thick across its tubular structure, cross-sectional descriptors cannot be provided for this species. Figure 2B,C show the loss of the external cell walls following pre-treatment with dilute acid. Figure 2B, in which *U. linza* was hydrolyzed in RO water, shows cell wall contortion as in Fig. 1B; again this contortion was not seen in 2 C when *U. linza* was hydrolyzed with seawater, with the cell walls retaining their original dimensions. Figure 2D,E show *U. linza* morphology following alkali pre-treatments prepared in RO water and seawater respectively. These have also ‘melded’ into flatter conglomerates, though residual cell structures have been retained in places, as seen in 2D. The cracks seen here were due to the SEM process rather than pre-treatment. Figure 2F (with RO water) and 2 G (with seawater) show the hydrothermal pre-treatments of *U. linza*, where the external cell wall retention for both images shows the process was less severe to the dilute acid pre-treatment (2B and 2 C).

**Figure 3:**
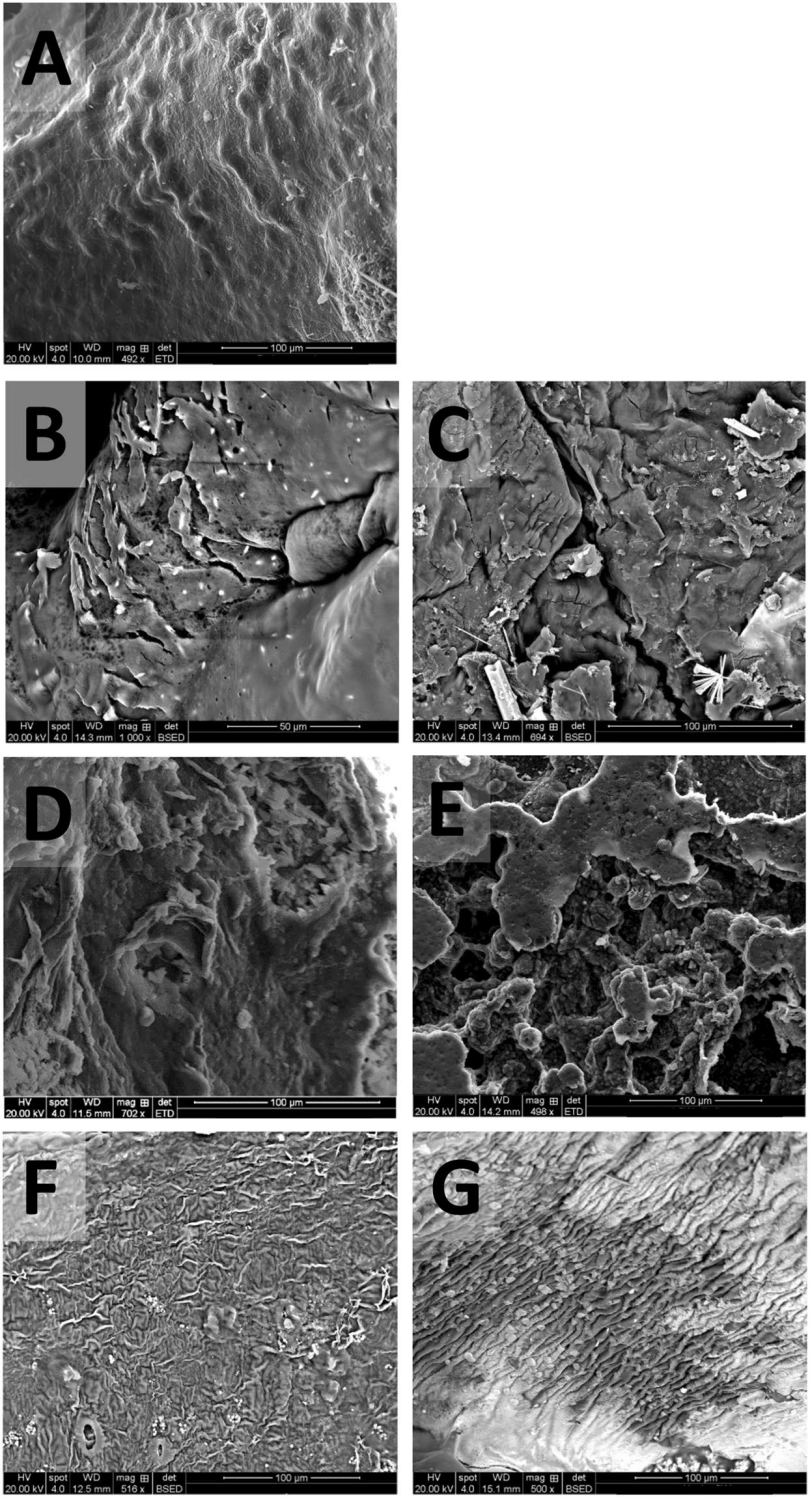
SEM images of *P. umbilicalis* following different thermochemical pre-treatments. (**A**) Freeze-dried sample; (**B**) dilute acid pre-treatment in RO water; (**C**) dilute acid pre-treatment in seawater; (**D**) alkaline pre-treatment in RO water; (**E**) alkaline pre-treatment in seawater; (**F**) hydrothermal pre-treatment in RO water; and (**G**) hydrothermal pre-treatment in seawater.

Figure 3 shows the effect of pre-treatments on *P. umbilicalis* tissues. Figure 3A shows typical surface conditions following freeze-drying. Figure 3B–E all show disintegration of the cellular structure following dilute acid and alkali pre-treatments, with only the hydrothermal pre-treatments retaining cellular integrity. For both hydrothermal pre-treatments in RO water (3 F) and seawater (3 G), the cell surface has become more convoluted than that in freeze-dried (3 A), but differences between the tissues were not clearly detected. For the *P. umbilicalis*, both dilute acid and alkali pre-treatments turned the material to small, disintegrated pieces. *P. umbilicalis* fronds are only one cell thick, so cellular wall loss on the external wall has led to the complete disintegration of the tissue. Such disintegration has been recorded in *Palmaria palmata*^16^, but this is the first-time that the disintegrations effect on a red seaweed species has been visualised using SEM. In comparison with *P. umbilicalis*, *U. linza* is comprised of a tube, with walls being monostromatic but once dried the two layers together provide additional support, therefore the cells were not disintegrated. *L. digitata* is several cells thick, so would not disintegrate by the dilute acid pre-treatment.

An alkaline pre-treatment disrupted the morphology for all macroalgae, with SEM images for all three species appearing as amorphous shapes, either in three dimensions (*L. digitata*, Fig. 1E), or a flatter, pitted surface (*U. linza*. Figure 2E; *P. umbilicalis* Fig. 3E). For this pre-treatment, the biomass was heated to 50 °C for 12 h in 5% NaOH solution. Though the temperature was lower than that used in the dilute acid (1% H_2_SO_4_, 121 °C, 30 min) or hydrothermal (121 °C, 15 min) pre-treatments, the higher proportion of ions present in alkali pre-treatment than that used for the dilute acid and longer residence time is likely to have caused this degradation. As alkaline removes the lignin whilst making the hemicellulose insoluble in lignocellulosic material, SEM images of eucalyptus structures following strong alkali pre-treatment shows a destruction of the fibre structure in the biomass, forming images similar to those seen in Fig. 1E^28^. Rice straw has been similarly imaged using SEM following NaOH pre-treatment and also resembles Fig. 1E. Corresponding analysis showed that a proportion of the cellulose and lignin was degraded^29^. Macroalgae, with high proportions of phenols and mixed-sugar polymers such as ulvan, is likely to be affected via a similarly mechanism.

### Pre-treatment of green seaweed with synthetic seawaters and salt solutions

In order to identify the component(s) in the seawater that are responsive for the improved seaweed hydrolysis, dilute acid pre-treatment using synthetic seawater and various salt solutions were investigated. Green seaweed was selected due to the relatively high sugar concentration obtained in the hydrolysis. Hydrolysis with 1 X synthetic seawater released 17.77 ± 0.51 g/L total sugars (Table 5) and use of double strength seawater (2X SW) released 18.69 ± 0.37 g/L total sugars. Experiment with individual salts revealed that use of 2.7%, 3.5% and 6% NaCl released 17.85 ± 0.41 g/L, 18.45 ± 0.78 g/L, and 21.33 ± 0.68 g/L sugars, respectively. The increase in sugars appeared to be related to the presence of ions in the liquid, as experiments with 3.5% MgSO_4_, 3.5% MgCl_2_ and 3.5% CaCl_2_ released 17.38 ± 0.33 g/L, 17.28 ± 0.11 g/L and 18.23 ± 0.33 g/L sugars (Table 5). All experiments with 3.5% NaCl, MgSO_4_, MgCl_2_ and CaCl_2_ led to a significant increase in sugars recovery when compared with that using RO water only (*p* = 0.001). Surprisingly experiment with 3.5% KCl failed to improve sugars releasing (16.86 ± 0.45 g/L, Table 5). Experiments with salt solutions analogous to their presence in seawater revealed that presence of 0.33% MgSO_4_ produced 16.8 ± 0.31 g/L, 0.25% MgCl_2_ produced 16.78 ± 0.08 g/L, 0.1% CaCl_2_ produced 16.80 g/L ± 0.28, 0.07% KCl produced 16.7 ± 0.28 g/L, respectively.

**Table 5:** The sugar composition of *U. linza* hydrolysates derived from 1% sulphuric acid pre-treatment in the presence of different salts.

	Glucose	Xylose	Arabinose	Galactose	Fucose	Rhamnose	Mannitol	Total sugars
**Sugars g/L**								
Acid pre-treatment water source								
RO water	8.16 ± 0.09	5.51 ± 0.08	2.27 ± 0.05	0.67 ± 0.03	0.09 ± 0.02	0.02 ± 0.01	0	16.74 ± 0.31
Seawater	8.81 ± 0.13	6.01 ± 0.07	2.54 ± 0.08	0.71 ± 0.03	0.08 ± 0.02	0.02 ± 0.01	0	18.20 ± 0.38
Synthetic	8.73 ± 0.09	5.85 ± 0.24	2.41 ± 0.08	0.68 ± 0.08	0.08 ± 0.09	0.02 ± 0.02	0	17.77 ± 0.51
Synthetic x 2	8.94 ± 0.11	6.23 ± 0.31	2.65 ± 0.04	0.77 ± 0.01	0.08 ± 0.12	0.04 ± 0.04	0	18.69 ± 0.37
2.7% NaCl	8.71 ± 0.05	5.86 ± 0.25	2.49 ± 0.11	0.69 ± 0.02	0.08 ± 0.02	0.02 ± 0.01	0	17.85 ± 0.41
3.5% NaCl	8.81 ± 0.07	6.11 ± 0.31	2.65 ± 0.09	0.78 ± 0.05	0.08 ± 0.03	0.02 ± 0.01	0	18.45 ± 0.78
6% NaCl	10.01 ± 0.1	7.33 ± 0.48	3.10 ± 0.25	0.79 ± 0.04	0.08 ± 0.01	0.02 ± 0.01	0	21.33 ± 0.68
0.33% MgSO_4_	8.21 ± 0.04	5.53 ± 0.15	2.28 ± 0.31	0.68 ± 0.07	0.08 ± 0.03	0.02 ± 0.01	0	16.80 ± 0.31
3.5% MgSO_4_	8.45 ± 0.12	5.80 ± 0.41	2.34 ± 0.15	0.68 ± 0.04	0.08 ± 0.02	0.02 ± 0.01		17.38 ± 0.33
0.25% MgCl_2_	8.21 ± 0.11	5.53 ± 0.15	2.25 ± 0.11	0.69 ± 0.09	0.08 ± 0.03	0.02 ± 0.01	0	16.78 ± 0.08
3.5% MgCl_2_	8.40 ± 0.21	5.78 ± 0.35	2.31 ± 0.10	0.69 ± 0.10	0.08 ± 0.02	0.02 ± 0.01	0	17.28 ± 0.11
0.1% CaCl_2_	8.19 ± 0.12	5.52 ± 0.21	2.30 ± 0.02	0.68 ± 0.04	0.09 ± 0.02	0.02 ± 0.01	0	16.80 ± 0.28
3.5% CaCl_2_	8.69 ± 0.22	6.11 ± 0.15	2.61 ± 0.11	0.72 ± 0.05	0.08 ± 0.02	0.02 ± 0.01	0	18.23 ± 0.33
0.07% KCl	8.16 ± 0.22	5.50 ± 0.02	2.25 ± 0.23	0.67 ± 0.04	0.08 ± 0.02	0.02 ± 0.01	0	16.70 ± 0.28
3.5% KCl	8.18 ± 0.31	5.55 ± 0.23	2.32 ± 0.24	0.71 ± 0.11	0.08 ± 0.02	0.02 ± 0.01	0	16.86 ± 0.45

An increase in hydrolysis yield with salts on lignocellulosic biomass has been reported previously^30,31^. Research has shown that increasing NaCl concentrations in a pre-treatment of sugar cane increased hydrolysis yield up to a threshold of around 1 M^32^; presence of seawater increased hydrolysis yields however, seawater only contains approximately 2.7% or 172 mM NaCl, indicating use of high salt concentrations would not be required during a pre-treatment.

### Conversion of seaweed hydrolysates to ethanol

The seawater based seaweed hydrolysis resulted in high sugar concentration in the hydrolysate. However, the hydrolysate contained high concentrations of salts, which may inhibit cell growth in the bioethanol fermentation process. In this study, a high salt tolerate marine yeast^22^, *Wickerhamomyces anomalus* M15, was used to convert the hydrolysate to ethanol. Seaweed hydrolysates obtained from 1% dilute acid pre-treatment were used as examples. As shown in Table 6, there were significantly higher ethanol concentrations in fermentations using hydrolysates produced using seawater than those produced using RO water. The final ethanol concentrations in the fermentations were 0.91 ± 0.21 (RO) and 1.41 ± 0.25 (SW) g/L with *L. digitata*; 4.12 ± 0.65 (RO) and 4.51 ± 0.21 (SW) g/L with *U. linza*; 4.23 ± 0.14 (RO) and 5.68 ± 0.51 (SW) g/L with *P. umbilicalis*, respectively (Table 6).

**Table 6:** Ethanol production in fermentation of seaweed hydrolysate using *Wickerhamomyces anomalus* M15. RO = reverse osmosis water, SW = seawater. N = 3, values show ± standard deviation.

Seaweed species	Ethanol concentration (g/L)
*L. digitata* (RO)	0.91 ± 0.21
*L. digitata* (SW)	1.41 ± 0.25
*U. linza* (RO)	4.12 ± 0.65
*U. linza* (SW)	4.51 ± 0.21
*P. umbilicalis* (RO)	4.23 ± 0.14
*P. umbilicalis* (SW)	5.68 ± 0.51

**Figure 4:**
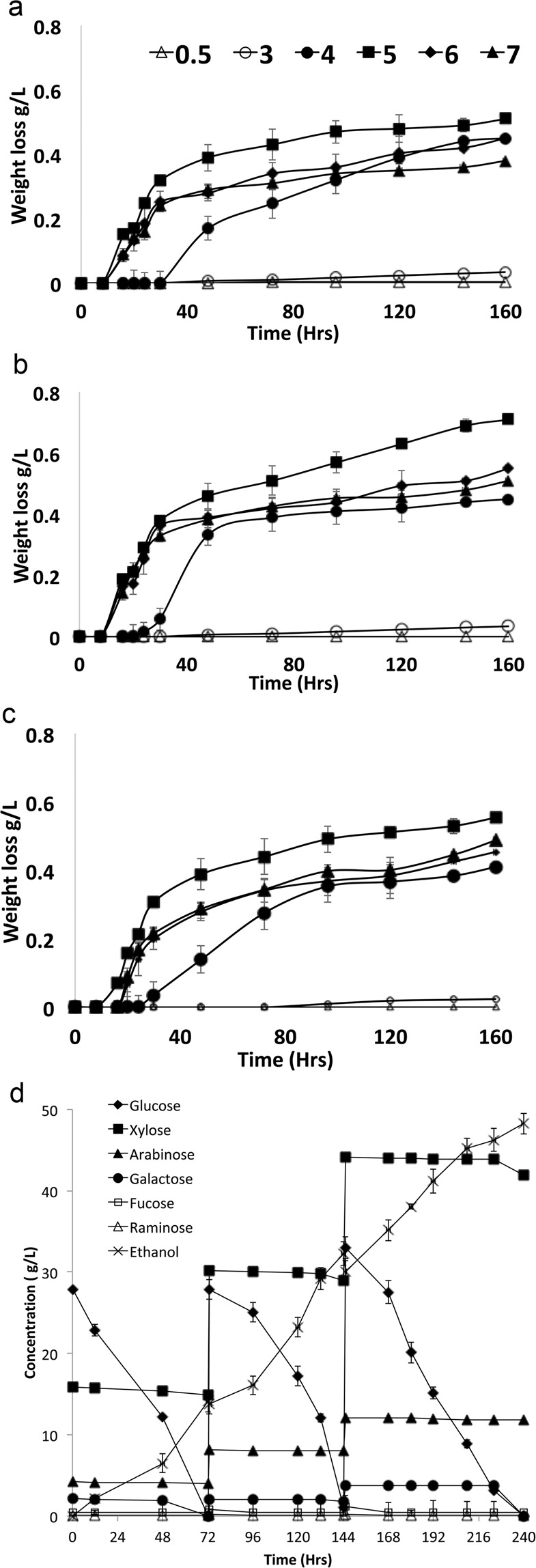
Performance of *W. anomalus* in fermentation of *U linza* hydrolysate at various starting pH’s (0.5–7) at (**a**) 25 °C, (**b**) 30 °C (**c**) 32 °C, (**d**) a fermentation using *W. anomalus* when fermenting a concentrated *U. linza* hydrolysate (**d**). Data representative of triplicate values N = 3.

Fermentations using hydrolysates derived from *U. linza* where further optimized by exploring the performance of *W. anomalus* M15 over a range of starting pH’s and incubation temperatures. Analysis revealed that this yeast preferred a starting pH of 5.0 with 30 °C as an optimal temperature (Fig. 4a–c).

To further increase bioethanol concentration, a seaweed hydrolysate derived from *U. linza* using 5% sulphuric acid and seawater was concentrated to produce a sugar-rich hydrolysate. The concentrated seaweed hydrolysate contained ~350 g/L total sugar with a sugar recovery yield 96.1%. As a consequence, the salinity of the medium was concentrated to 16.6 ± 0.8%. The concentrated hydrolysate was then diluted in seawater to give a starting sugar load of approximately 50 g/L (27.9 g/L glucose, 2.1 g/L galactose, 15.21 g/L xylose, 4.1 g/L arabinose, 0.4 g/L fucose, and 0.1 g/L rhamnose, salinity ~ 5%). This sugar mixture was adjusted to pH 5.0 and then fermented by *W. anomalus* M15. The progress of the fermentation was determined by measuring the presence of glucose in the fermentation. After available glucose had been used by the yeast in the fermentation (72 hours), an additional 50 g/L total sugar mix was added, with a further 50 g/L total sugar mix added after 144 hours. During the fermentation, glucose was the principal sugar used by the yeast as expected. Only when glucose was depleted, galactose was utilized (Fig. 4d). During the fermentation, approximately 5.52 ± 0.12 g/L xylose and 1.76 ± 0.13 g/L arabinose were depleted. Fucose and rhamnose were not utilized by *W. anomalus* M15 during the fermentation.

In the first 72 hours of the fermentation, 14.10 ± 0.13 g/L ethanol was produced with a 0.46 ± 0.04 g/g conversion efficiency and a 0.196 ± 0.010 g/L/h productivity. At 144 hours, 30.28 ± 0.03 g/L ethanol was produced, and a final ethanol concentration of 48.24 ± 0.07 g/L ethanol was produced from 95.52 ± 0.08 g/L sugars consumed after 240 hours. The ethanol conversion efficiency was 0.495 ± 0.010 g/g consumed sugars, while the overall productivity was 0.201 ± 0.005 g/L/h. Based on this result, the overall ethanol production yield was estimated to be 0.329 g/g available sugars in the seaweed, equivalent to 0.096 g/g dry weight seaweed. Interestingly the amount of ethanol produced (48.24 g/L) was higher than the theoretical maximum (0.51 g/g) possible from the conversion of glucose and galactose, indicating that the yeast converted other substrates possibly the available pentose sugars into ethanol. The sugar conversion yield (0.495 g/g) achieved in this study was higher than recent reports where 0.38–0.44 g ethanol per g pentose sugars was obtained^33,34^. The overall ethanol yield (0.096 g/g seaweed) agreed with similar studies using green seaweed, where 0.092–0.12 g ethanol per g seaweed was reported^35,36^.

In order to make the process economically feasible, ethanol concentration in the fermentation broth should reach at least 4–5%^37^. In this study, 48.24 g/L ethanol was obtained, which is the highest ethanol titres reported to date using green seaweed^15^, although 64.3 g/L of ethanol has been produced from a hydrolysate derived from a red seaweed *K. alvarezii*^38^. In order to improve bioethanol concentration in the fermentation broth, the initial sugar concentrations in the hydrolysate has to be enhanced. This could be achieved by (i) selecting a seaweed species with high total carbohydrate content^39,40^; (ii) improving the solid loading ratio in the hydrolysis process^40–42^; (iii) concentrating the hydrolysate e.g. using rotary evaporation^38^; or (iv) integrating ethanol production with *in-situ* seaweed saccharification. In this study, a rotary evaporation process was used to concentrate the seaweed hydrolysate to a total sugar concentration of ~350 g/L. The ethanol concentration could be further improved if additional sugars were supplied. The selection of a high tolerant microorganism, such as *W. anomalus* M15, for high titre fermentation is crucial. *W. anomalus* M15 was demonstrated to tolerate up to 21.4% (w/w) sodium chloride, 49.6 mM furfural and 167.8 mM acetic acid^22^. Therefore, although the salt content reached 5% in the fermentation using a medium prepared with concentrated seaweed, no inhibitory effect was observed.

## Conclusions

In this study, seawater instead of RO water has been used for the pre-treatment of brown, green and red seaweed. Significant improvement of sugar recovery yield was observed in all seaweed species, both due to the higher total sugar concentration in hydrolysate and higher liquid recovery ratio. The highest hydrolysis yields obtained for brown, green and red seaweed were 64.6%, 69.1% and 63.0%, respectively, in hydrolysis using 5% sulphuric acid. Fermentations using concentrated green seaweed hydrolysate (*U. linza*) resulted in 48.2 g/L ethanol, equivalent to an overall yield of 0.329 g/g available sugar in the seaweed, 0.096 g/g dry weight seaweed.

## Supplementary information

Supplementary Figure S1.

## Data Availability

All data is available upon request.
