# Supplementary figures and images for: The utilization of seawater for the hydrolysis of macroalgae and subsequent bioethanol fermentation
Source: Sci Rep. 2020 Jun 16;10:9728. doi: 10.1038/s41598-020-66610-9 (PMC7297732; doi:10.1038/s41598-020-66610-9)

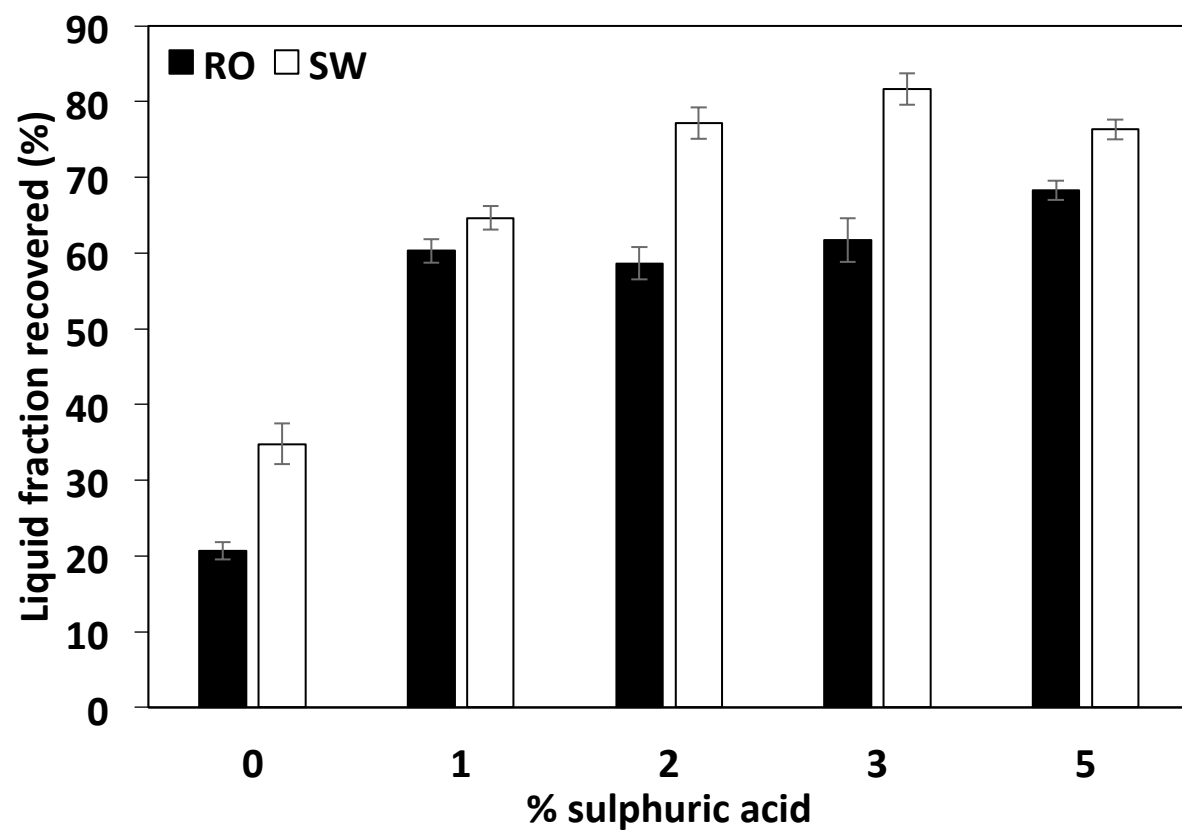

Supplement: Supplementary file 1 — Supplementary Figure S1. [file 41598_2020_66610_MOESM1_ESM.pdf]
